# Baseline peripheral neuropathy was associated with age and a prognostic factor in newly diagnosed multiple myeloma patients

**DOI:** 10.1038/s41598-022-13935-2

**Published:** 2022-06-16

**Authors:** Mengmeng Dong, Jinna Zhang, Xiaoyan Han, Jingsong He, Gaofeng Zheng, Zhen Cai

**Affiliations:** 1 The First Affiliated Hospital, School of Medicine, Zhejiang University, Bone Marrow Transplantation Center, No.79 Qingchun Rd, Zhejiang 310003 Hangzhou, China; 2 Institute of Hematology, Zhejiang University, Hangzhou, 310003 Zhejiang China

**Keywords:** Myeloma, Neurological disorders

## Abstract

Multiple myeloma (MM) is an incurable plasma cell hematological malignancy. Bortezomib has become the primary drug in the treatment of patients with MM. However, its negative effects, especially peripheral neuropathy (PN), affect the patients’ life quality and treatment continuity. However, there are few studies on baseline PN of MM, and little is known of the impact of baseline PN on the prognosis of MM patients. Therefore, we reviewed the clinical data of newly diagnosed MM patients in our center, explored the influencing factors of baseline PN, and evaluated PN’s influence on the prognosis of MM patients undergoing induction therapy with bortezomib. According to the inclusion and exclusion criteria, 155 MM patients were eligible for the retrospective study. The multivariate regression analysis, generalized additive fitting smooth curve, the receiver operating characteristic curve (ROC) and K-M curve were conducted in this study. We found that baseline PN in patients with MM was age-related; MM patients with baseline PN have more severe bortezomib induced PN (BiPN) during the four courses of induction therapy with bortezomib as the primary regimen and worse PN outcome after induction therapy. Additionally, the progression-free survival (PFS) and overall survival (OS) of MM patients with baseline PN were worse than those of the MM patients without baseline PN.

## Introduction

Multiple myeloma (MM) is an incurable malignant plasma cell tumor, and the incidence is second-highest in hematological malignancies^1^. The monoclonal immunoglobulin secreted by malignant plasma cells causes several clinical symptoms, such as anemia, hypercalcemia, kidney damage, bone pain, etc.^2^. MM is nearly 1.8% of all newly diagnosed cancers, and the percentage of estimated deaths in MM of all cancer deaths is almost 2.0%^3^. The median age at diagnosis is 70 years, and two-thirds of MM patients are more than 65 years old at first diagnosis^2^. Moreover, advanced age is associated with poor clinical prognosis^4,5^. Every year, about five of 100,000 people suffer from MM, and the overall prognosis has been relatively unfavorable until the application of new therapeutic strategies, such as proteasome inhibitor, immunomodulator and chimeric antigen receptor T-cell (CART) therapy. The effectiveness and prognosis of MM have been greatly improved^6^. Survival with tumors and chronic disease management have gradually become new concepts in the treatment and management of MM patients. Improving the life quality and happiness index of MM patients is an important goal for the treatment of MM patients^7,8^.

Peripheral neuropathy (PN) is an essential factor that affects the life quality of MM patients^9^. Many patients develop symptoms of PN at the initial stage of the disease mainly manifested as numbness of the limbs, tingling, autonomic nerve damage symptoms, and even small motor dysfunction^9^. There are many mechanisms to stimulate PN in MM patients. Monoclonal protein (M-protein), light chain immunoglobulin in nerve cells (amyloidosis), or medulla compression can directly damage nerve cells^10^. Additionally, the high blood viscosity due to high M-protein levels will slow down the blood flow, leading to neurological symptoms^11^.

In addition to the PN caused by MM disease itself, treatment-related PN has also been paid attention to. Bortezomib has now become the basic drug in the treatment of MM patients^12^. Although bortezomib is one of the most widely used for MM, it can induce several adverse events. One of its side effects is PN, which affects the patients’ life quality, compliance, treatment tolerance, and continuity^13^.

There are few studies on the relationship between age and baseline PN in MM patients. And we know little about the association between baseline PN and the prognosis of MM patients. Therefore, we reviewed the clinical data of patients with newly diagnosed MM in our department, explored the influencing factors of baseline PN, and evaluated the impact of baseline PN on the clinical prognosis of bortezomib-based treatment. Our research paid attention to the PN of MM patients in the early stage, and provide real-world data support for MM patients to choose an individualized treatment plan that can maintain a better life quality.

## Methods

This retrospective cohort study collected clinical data of newly diagnosed multiple myeloma (MM) patients from the First Affiliated Hospital of Zhejiang University School of Medicine from March 2011 to October 2015. Ethics approval and consent to participate.

The selection criteria were as follows: (1) newly diagnosed MM patients in our unit from March 2011 to October 2015; (2) the diagnosis of MM was based on the International Myeloma Working Group (IMWG) symptomatic MM standard^14^; (3) they all had blood or urine monoclonal protein that could be evaluated for curative effect; (4) the MM patients have data of PN at baseline, and each course of induction therapy; (5) appropriation and agree to use bortezomib-based combination chemotherapy as the first-line induction treatment plan; (6) the follow-up data was recorded in our unit until lost or death; (7) according to the above criteria, 361 MM patients were excluded due to missing primary data, 47 patients excluded due to no treatment, 83 patients excluded on account of not completed 4 courses of bortezomib-based regimen therapy in our center, 95 patients excluded due to previous other neuropathy, and 31 patients excluded because of serious heart, lung, liver or/and kidney disease or other tumors. As a result, 155 patients were selected from 772 MM patients in the center during the above time for this study.

The diagnosis of PN was mainly based on the clinical manifestations of the patients, physical examination and electromyography. The details were as follows: (1) A clear history of MM; (2) Abnormalities in any one or more of the following four aspects of neurological examination: ① physical examination of sensory nerve (pain, temperature, touch, vibration, etc.); ② physical examination of motor nerve (muscle strength, ankle reflex, radial Reflex, etc.); ③ electromyography showed one or more nerve slow down. Physical examination was performed by the responsible physician and nurse. Electromyography was performed by the examining doctor. The above information was collected by a physician and reviewed by another physician.

At the end of each cycle of the four inductive treatments, the patients were assessed their effectiveness as complete remission (CR), very good partial remission (VGPR), partial remission (PR) or stable disease (SD), according to NCCN Guidelines Insights: Multiple Myeloma, Version 1.2020.

### Data collection

Baseline demographic and clinical data of the MM patients were collected by electronic retrieval from medical records, and we conducted a retrospective review. The following indicators were evaluated: (1) Demographic characteristics, including age and gender; (2) clinical data, including immunoglobin type, light chain type, Durie-Salmon stage (DS stage), International Staging System (ISS stage), baseline PN, albumin, serum creatinine, β2-microglobin (β2-MG), lactate dehydrogenase (LDH), and hemoglobin (HB); (3) administration method and dosage of bortezomib; (4) the best curative effectiveness within four courses of induction therapy; (5) the degree of PN induced by bortezomib after treatments; (6) follow-up in our unit death or loss to follow-up; (7) progression-free survival (PFS) time and overall survival (OS) time.

The diagnosis of MM was based on the new IMWG symptomatic MM criteria^14^. And the effectiveness evaluation used the MM National Comprehensive Cancer Network clinical practice guidelines 2020^15^, and the PN grade was according to the National Cancer Institute Common terminology criteria for adverse events (CTCAE 4.03, Supplementary Table 1).

### Statistical analysis

Continuous data were expressed as mean ± standard deviation. Categorical variables were expressed as numbers or percentages. Student’s *t*-test and Chi-square test were used to assess the difference between two groups.

First, we conducted a univariate analysis of the newly diagnosed MM patients’ PN and potentially related factors. We found that the patients’ age was related to the grade of baseline PN. Then, variable-adjusted binary Logistic regression model and generalized addition fitting smooth curve analyzed the relationship between age and baseline PN. The receiver operating characteristic curve (ROC) assesses the predictive value of age for baseline PN in newly diagnosed MM patients.

Further variable-adjusted multiple linear regression was used to study the influence of the baseline PN and the outcome of bortezomib induced PN (BiPN, assessed 6 months after induction therapy) in MM patients, and a violin plot was used to show their relationship. Finally, variable-adjusted COX proportional hazards regression model analysis and K-M curve was used to analyze the relationship between the baseline PN and PFS/OS in MM patients.

In statistical analysis, the best curative effectiveness ≤ complete remission (CR) was labelled as 1, very good partial remission (VGPR) as 2, partial remission (PR) as 3, stable disease (SD) as 4. All probabilities were two-tailed. Empower Stats (www.empowerstats.com; X & Y Solution, Inc., Boston, MA, USA), R software (http://www.R-project. organization) and GraphPad Prism 8 were used for statistics and graphing. A p value of < 0.05 was considered significant.

### Ethics approval and consent to participate

Reference number (IIT20210611A) from Ethical Inspection of the First Affiliated Hospital, College of Medicine, Zhejiang University. Informed consent was waived by the Clinical Research Ethics Committee of the First Affiliated Hospital, College of Medicine, Zhejiang University, because the medical records used in this study were obtained from previous clinical diagnosis and treatment. Exemption from informed consent will not adversely affect the rights and health of subjects. All methods were performed in accordance with the Declaration of Helsinki.

## Results

**Figure 1:**
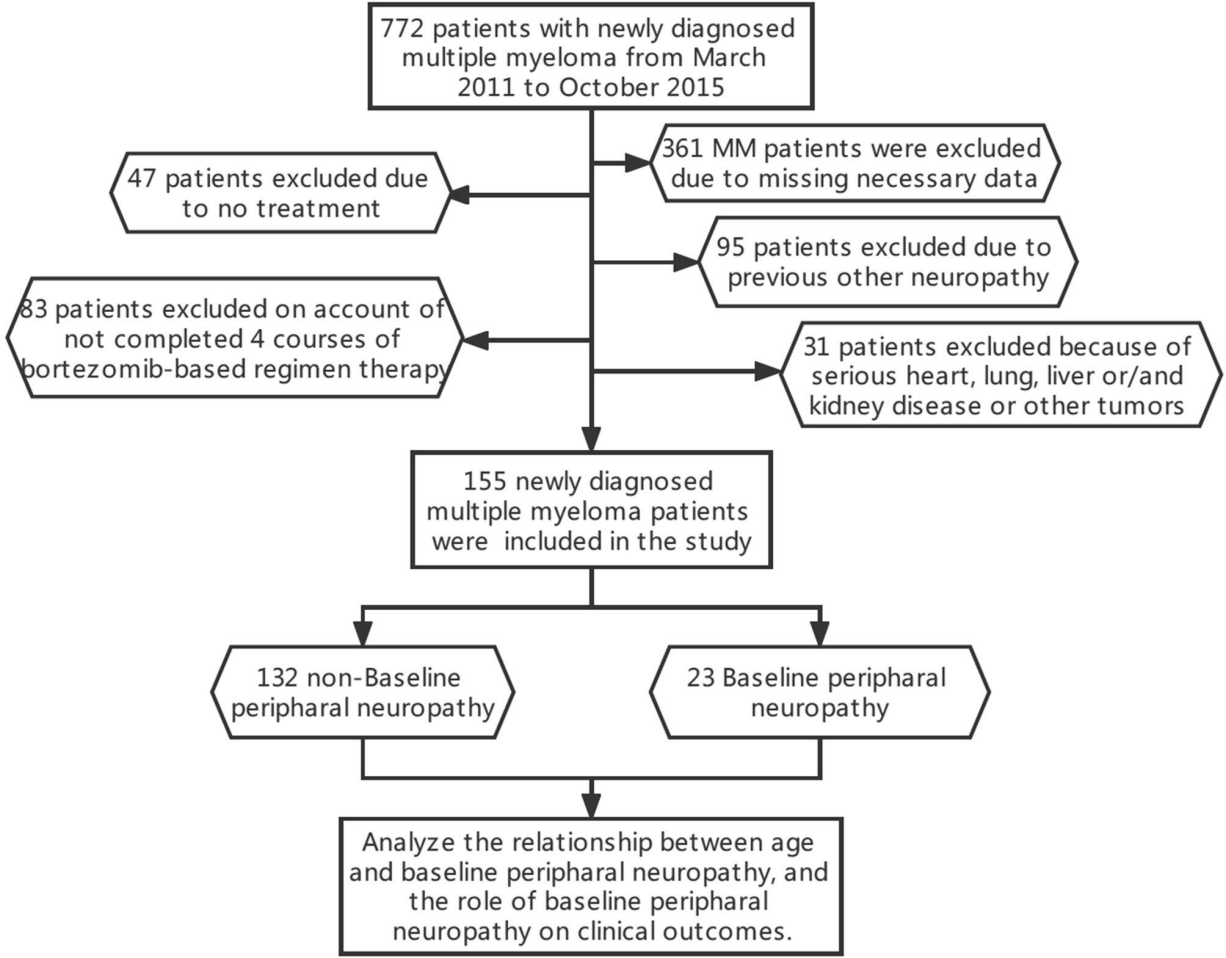
Flow chart of participants (created by Processon, https://www.processon.com/). Seven hundred and seventy-two patients participated in the retrospective study. After exclusion, 155 patients were included according to the criteria.

Among the 772 newly diagnosed MM patients treated in our unit from March 2011 to May 2015, 617 patients were excluded after the screening process of Fig. 1, and 155 newly diagnosed MM patients were included in this study.

### Age was independently associated with baseline PN in MM patients

Among the 155 MM patients, 23 had baseline PN, and 132 had no baseline PN. There was no significant difference in immunoglobulin typing, light chain typing, DS staging and ISS staging between the two groups (Table 1).

**Table 1:** Description of clinical data.

Variable	Non-baseline PN	Baseline PN	P-value	Variable	Non-baseline PN	Baseline PN	P-value
N	132	23		LDH (g/L)	207.60 ± 106.80	191.09 ± 60.58	0.483
Age	59.96 ± 9.49	66.00 ± 6.61	0.003	HB (g/L)	91.93 ± 24.14	98.23 ± 22.33	0.244
Male	78 (59.54%)	11 (47.83%)	0.281	Bortezomib dose			0.423
BMI	22.55 ± 2.59	22.04 ± 2.84	0.399	BTZ 1.0 mg/m^2^	57 (43.18%)	12 (52.17%)	
**Immunoglobin type**			0.862	BTZ 1.3 mg/m^2^	75 (56.82%)	11 (47.83%)	
NA	41 (31.30%)	5 (21.74%)		Administration route			0.781
IgG	50 (38.17%)	10 (43.48%)		IV	73 (55.30%)	12 (52.17%)	
IgA	36 (27.48%)	7 (30.43%)		SC	59 (44.70%)	11 (47.83%)	
IgD	3 (2.29%)	1 (4.35%)		The most severe BiPN			0.007
IgG &IgA	1 (0.76%)	0 (0.00%)		0	44 (33.33%)	0 (0.00%)	
**Light chain type**			0.666	1	63 (47.73%)	14 (60.87%)	
NA	17 (12.98%)	4 (17.39%)		2	23 (17.42%)	8 (34.78%)	
λ	64 (48.85%)	9 (39.13%)		3	2 (1.52%)	1 (4.35%)	
κ	50 (38.17%)	10 (43.48%)		PN recovery			0.002
**DS stage**			0.666	0	42 (31.82%)	1 (4.35%)	
I	9 (6.87%)	1 (4.35%)		1	89 (67.42%)	20 (86.96%)	
II	11 (8.40%)	3 (13.04%)		2	1 (0.76%)	2 (8.70%)	
III	111 (84.73%)	19 (82.61%)		BE			0.039
**ISS stage**			0.082	1	62 (46.97%)	8 (34.78%)	
1	36 (27.48%)	11 (50.00%)		2	54 (40.91%)	7 (30.43%)	
2	47 (35.88%)	4 (18.18%)		3	12 (9.09%)	5 (21.74%)	
3	48 (36.64%)	7 (31.82%)		4	4 (3.03%)	3 (13.04%)	
Creatinine (μmol/L)	121.73 ± 145.51	117.69 ± 118.79	0.555	Relapse	94 (71.21%)	18 (81.82%)	0.301
≥ 177 (μmol/L)	16 (12.12%)	5 (21.74%)	0.214	PFS (month)	34.07 ± 27.15	19.48 ± 17.33	0.016
Albumin (U/L)	38.28 ± 8.16	38.80 ± 7.19	0.772	Mortality	87 (66.41%)	15 (68.18%)	0.871
β2-MG (mg/L)	5.99 ± 5.29	4.56 ± 2.68	0.209	OS (month)	49.64 ± 31.26	28.56 ± 18.81	0.003

The best curative effectiveness ≤ complete remission (CR) was labelled as 1, very good partial remission (VGPR) as 2, partial remission (PR) as 3, stable disease (SD) as 4.

*BMI* body bass index, *β2-MG* β2 microglobin, *LDH* lactate dehydrogenase, *HB* haemoglobin, *PN* peripheral neuropathy, *IV* intravenous injection, *SC* Subcutaneous injection, *BiPN* the most severe bortezomib induced PN during the inductive therapy, *BE* the best efficacy during the inductive, *PFS* Progression-free survival, *OS* overall survival.

Univariate analysis was used to estimate the correlation between the age of MM patients baseline PN (OR 1.08, *p* = 0.0053). OR was slightly greater than 1, in spite of the low p-value. Furthermore, taking age as the independent variable, after adjusting the gender, body mass index (BMI), immunoglobin type, light chain type, DS stage, creatinine, β2-microglobulin (β2-MG), and lactate dehydrogenase (LDH), the results of the fully adjusted model showed that age was independently related to PN (OR 1.11, 95% CI (1.03, 1.18), p = 0.0044). As the age varied from 31 to 82 years old, in order to analyze the effect of the age on baseline PN in stratify, the patients were then divided into two groups based on 60, 65, and 70 years, respectively. The binary Logistic regression adjusted by potential factors showed that baseline PN incidence was higher in the high-age group than in the low-age group in all these three models. Among them, patients ≥ 60 years old compared with patients < 60 years old, OR 3.58, 95% CI (1.14, 11.18), *p* = 0.0284; patients ≥ 65 years old compared with patients < 65 years old, OR 3.92, 95% CI (1.35, 11.38), *p* = 0.0120. MM patients aged ≥ 70 years old had more severe baseline PN compared with patients < 70 years old, OR 3.15, 95% CI (0.91, 10.87). However, *p* value was 0.0695, more than 0.05 (Table 2).

**Table 2:** Variable-adjusted binary Logistic regression analysis between age and the baseline PN.

Variable	non-baseline PN (N)	baseline PN (N)	Crude model (OR, 95% CI, P)	Minimally adjusted model (OR, 95% CI, P)	Fully adjusted model (OR, 95% CI, P)
Age	132	23	1.08 (1.02, 1.14) 0.0053	1.09 (1.03, 1.15) 0.0048	1.10 (1.03, 1.18) 0.0044
**Age (year)**					
< 60	71 (53.79%)	6 (26.09%)			
≥ 60	61 (46.21%)	17 (73.91%)	3.30 (1.22, 8.89) 0.0183	3.40 (1.19, 9.73) 0.0222	3.58 (1.14, 11.18) 0.0284
**Age (year)**					
< 65	99 (75.00%)	11 (47.83%)			
≥ 65	33 (25.00%)	12 (52.17%)	3.27 (1.32, 8.11) 0.0105	3.50 (1.34, 9.15) 0.0107	3.92 (1.35, 11.38) 0.0120
**Age (year)**					
< 70	114 (86.36%)	17 (73.91%)			
≥ 70	18 (13.64%)	6 (26.09%)	2.24 (0.78, 6.42) 0.1351	2.62 (0.82, 8.42) 0.1049	3.15 (0.91, 10.87) 0.0695

Statistical method: variable-adjusted binary Logistic regression.

Crude model adjust for: None.

Minimally adjusted model adjust for: gender, immunoglobin type, light chain type.

Fully adjusted model adjust for: gender, BMI, immunoglobin type, light chain type, DS stage, Creatinine, β2 microglobulin, lactate dehydrogenase.

*PN* peripheral neuropathy.

**Figure 2:**
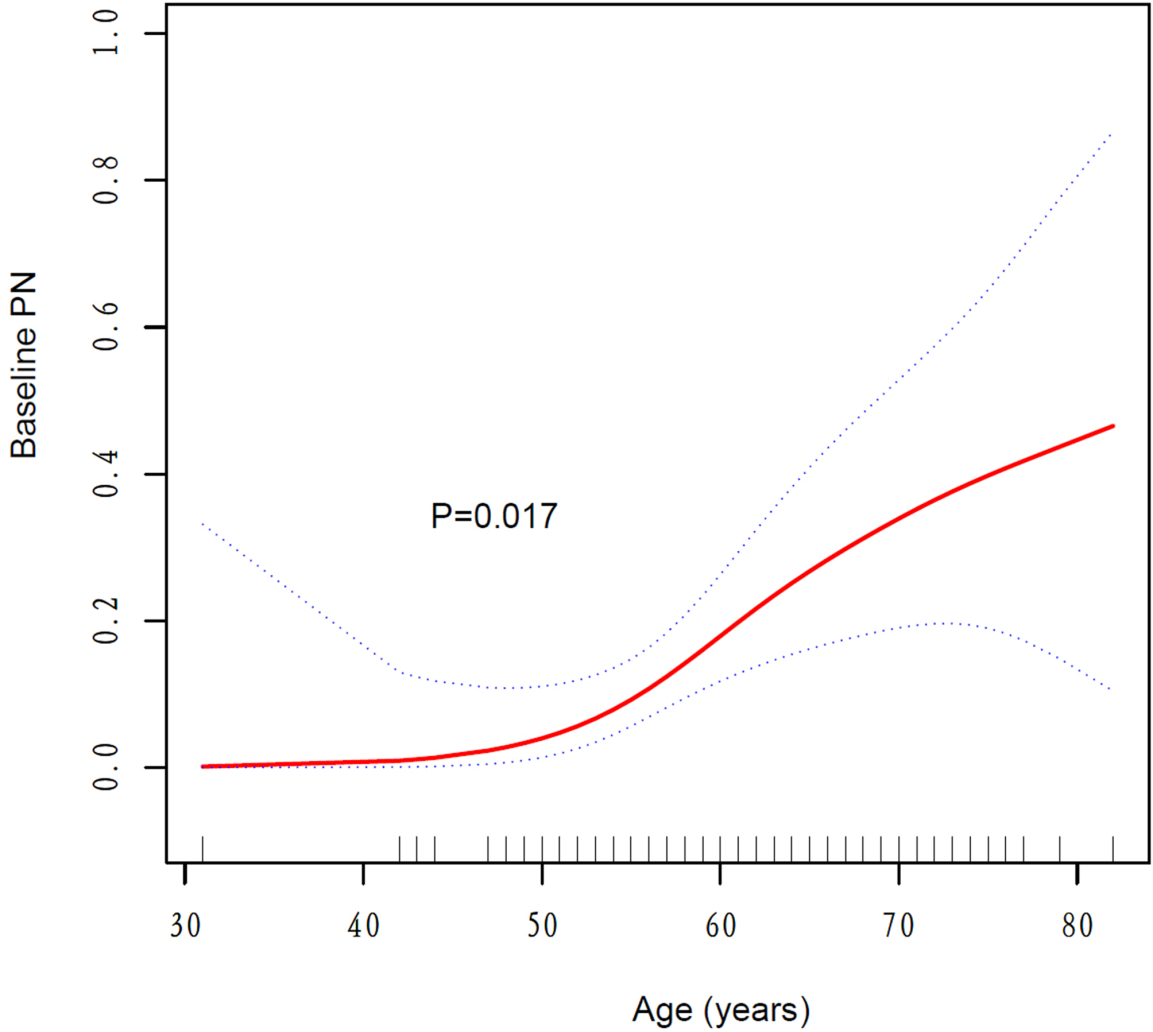
Spline smoothing curve for the cross-sectional correlation between age and baseline peripheral neuropathy (*p* = 0.017, created by Empower Stats and R software, www.empowerstats.com; http://www.R-project). Generalize additive models was used in the analysis, the red line represented the fitted relationship between age and baseline PN, and the blue lines referred the 95% CI. The model was adjusted for: gender, immunoglobin type, light chain type, DS stage, creatinine, β2 microglobulin; lactate dehydrogenase. *PN* peripheral neuropathy.

**Figure 3:**
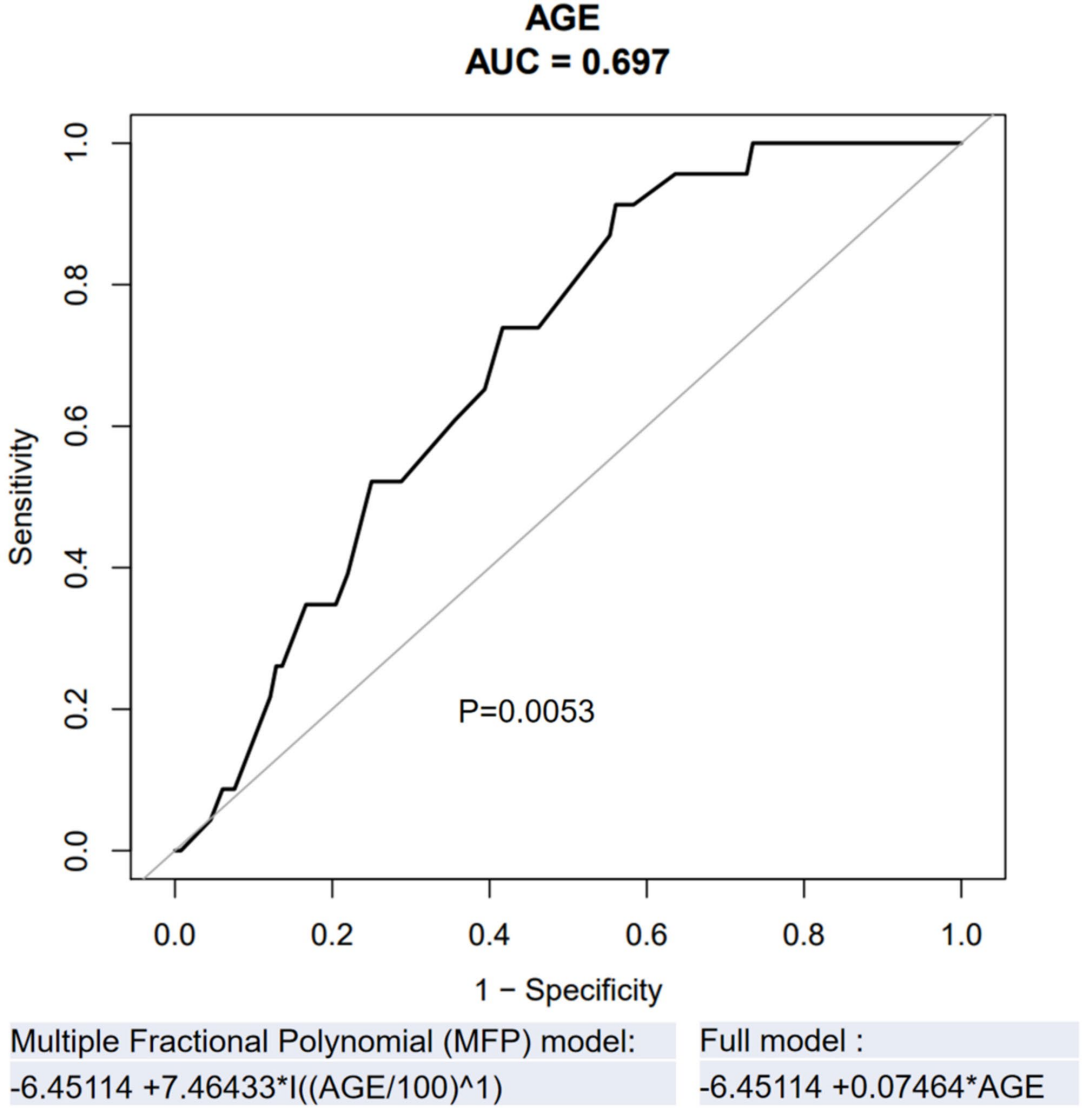
Predictive model and the ROC curve (created by Empower Stats and R software, www.empowerstats.com; http://www.R-project) analysis for age and baseline peripheral neuropathy (AUC = 0.697; *p* = 0.0053). Multiple Fractional Polynomial (MFP) model: − 6.45114 + 7.46433 × I((AGE/100)^1^). Full model: − 6.45114 + 0.07464 × AGE.

The generalized additive fitting smooth curve fitting showed that age was related to the baseline PN of MM patients. The older the age, the greater the likelihood of baseline PN (Fig. 2). The analysis of the ROC curve showed that the area under curve was 0.697, *p* = 0.0053. This result showed that age had a certain predictive effect on baseline PN in MM patients (Fig. 3).

### MM patients with baseline PN had more severe BiPN and worse PN outcome

These MM patients all conducted induction therapy with the bortezomib-based regimen. During the treatment, the bortezomib-induced peripheral neuropathy (BiPN), the best curative effectiveness, and the PFS/OS were observed and recorded (Table 1).

**Figure 4:**
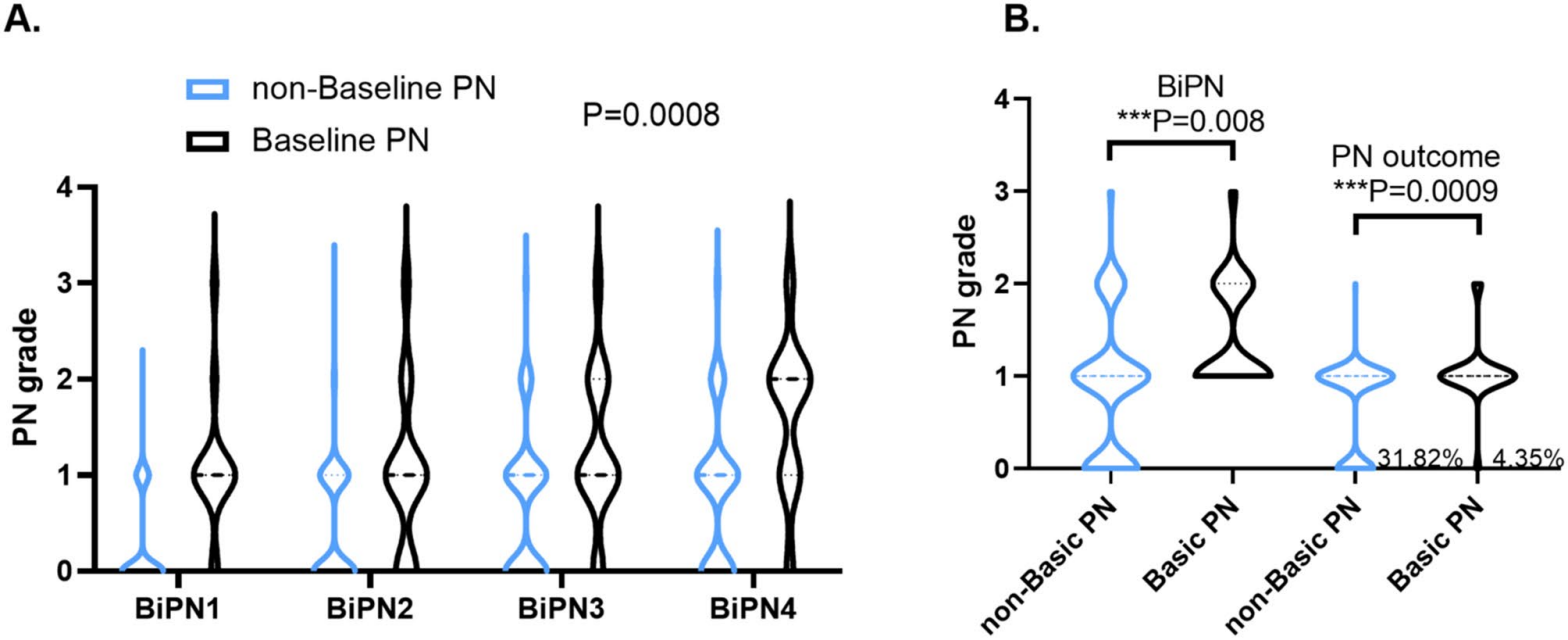
Violin plot showed PN grades between different baseline PN groups (created by GraphPad Prism 8). (**A**) MM patients with baseline PN suffered more severe bortezomib-induced peripheral neuropathy (BiPN) in bortezomib-based inductive regimen (*p* = 0.0008). PN1, PN2, PN3, and PN4 were acronyms for BiPN in cycles one, two, three, and four, respectively. (**B**) The grade of the most severe BiPN was higher in MM patients with baseline PN. PN outcome was assessed 6 months after induction therapy. MM patients suffered more severe PN after induction therapy (*p* = 0.0009). *MM* multiple myeloma, *PN* peripheral neuropathy.

Univariate analysis showed that baseline PN of MM patients was significantly correlated with the severity of BiPN (*p* = 0.0008). The violin plot showed BiPN grades between different baseline PN groups in induction therapy. MM patients with baseline PN suffered more severe BiPN in every cycle in bortezomib-based inductive regimen (*p* = 0.0008, Fig. 4A), with fewer BiPN of grade 1 and more 2/3 grades. The most severe BiPN during induction therapy was also observed and recorded. After adjusting the age, gender, BMI, immunoglobin type, light chain type, DS stage, creatinine, β2-MG, LDH, bortezomib dose, and administration route, the variable-adjusted linear regression showed that patients with baseline PN had more severe BiPN than patients without baseline PN (β = 0.52, 95% CI (0.18, 0.86), *p* = 0.0037, Table 3). After treatment, some patients with BiPN would be relieved. PN assessed 6 months after induction therapy was recorded as PN outcome. The variable-adjusted linear regression adjusted for potential factors showed that patients with baseline PN had worse PN outcome than those without baseline PN (β = 0.34, 95% CI (0.12, 0.56), *p* = 0.0034, Table 3). The violin plot intuitively showed that MM patients with baseline PN had more severe BiPN (*p* = 0.0008, Fig. 4A) and worse PN outcome (*p* = 0.0009, Fig. 4B).

**Table 3:** Mutiple regressions analysis between the most severe BiPN/PN outcome/BE/PFS/OS and the baseline PN.

Variable	Crude model (β, 95% CI, P)	Minimally adjusted model (β, 95% CI, P)	Fully adjusted model (β, 95% CI, P)
**The most severe BiPN**^**1**^			
Non-baseline PN	0	0	0
Baseline PN	0.56 (0.24, 0.88) 0.0008	0.51 (0.16, 0.85) 0.0044	0.52 (0.18, 0.86) 0.0037
**PN outcome**^**1**^			
Non-baseline PN	0	0	0
Baseline PN	0.35 (0.15, 0.56) 0.0010	0.36 (0.14, 0.57) 0.0016	0.34 (0.12, 0.56) 0.0034
**BE**^**1**^			
Non-baseline PN	0	0	0
Baseline PN	0.45 (0.09, 0.81) 0.0158	0.33 (− 0.03, 0.69) 0.0774	0.26 (− 0.12, 0.63) 0.1796
Variable	Crude model (HR, 95% CI, P)	Minimally adjusted model (HR, 95% CI, P)	Fully adjusted model (HR, 95% CI, P)
**PFS**^**2**^			
Non-baseline PN	1	1	1
Baseline PN	2.11 (1.26, 3.53) 0.0045	1.97 (1.15, 3.36) 0.0130	2.05 (1.17, 3.59) 0.0122
**OS**^**2**^			
Non-baseline PN	1	1	1
Baseline PN	1.90 (1.09, 3.33) 0.0244	1.81 (1.01, 3.22) 0.0447	1.90 (1.04, 3.47) 0.0381

Crude model adjust for: None.

Minimally adjusted model adjust for: gender, immunoglobin type, light chain type.

Fully adjusted model adjust for: age, gender, immunoglobin type, light chain type, DS stage, Creatinine, β2 microglobulin; lactate dehydrogenase; bortezomib dose and administration route.

*β* regression coefficient, *PN* peripheral neuropathy, *BiPN* bortezomib induced PN, *BE* the best efficacy during the inductive therapy, *PFS* Progression-free survival, *OS* overall survival.

Statistical methods: ^1^variable-adjusted multiple linear regression; ^2^variable-adjusted COX proportional hazards regression.

### PFS and OS were worse in MM patients with baseline PN than those without baseline PN

The curative effectiveness was a concern for both MM patients and clinicians. At the end of each cycle of the four induction treatments, the effectiveness was assessed: complete remission (CR), very good partial remission (VGPR), partial remission (PR) or stable disease (SD), according to NCCN Guidelines Insights—Multiple Myeloma, Version 1.2020. After completing induction therapy, the best effectiveness (BE) of these four cycles was evaluated for the following analysis. The results of the univariate analysis showed that MM patients with baseline PN had worse BE (β = 0.45 95% CI (0.15, 0.56), *p* = 0.015). After adjusting potentially related multiple factors, the fully adjusted model of variable-adjusted linear regression showed that baseline PN was not independently correlated with the BE (β = 0.26 95% CI (− 0.12, 0.63), *p* = 0.1796, Table 3). The result showed that baseline PN might be associated with BE, however, not independently. The best effectiveness of the induction therapy for MM patients with baseline PN was worse than that of patients without baseline PN.

**Figure 5:**
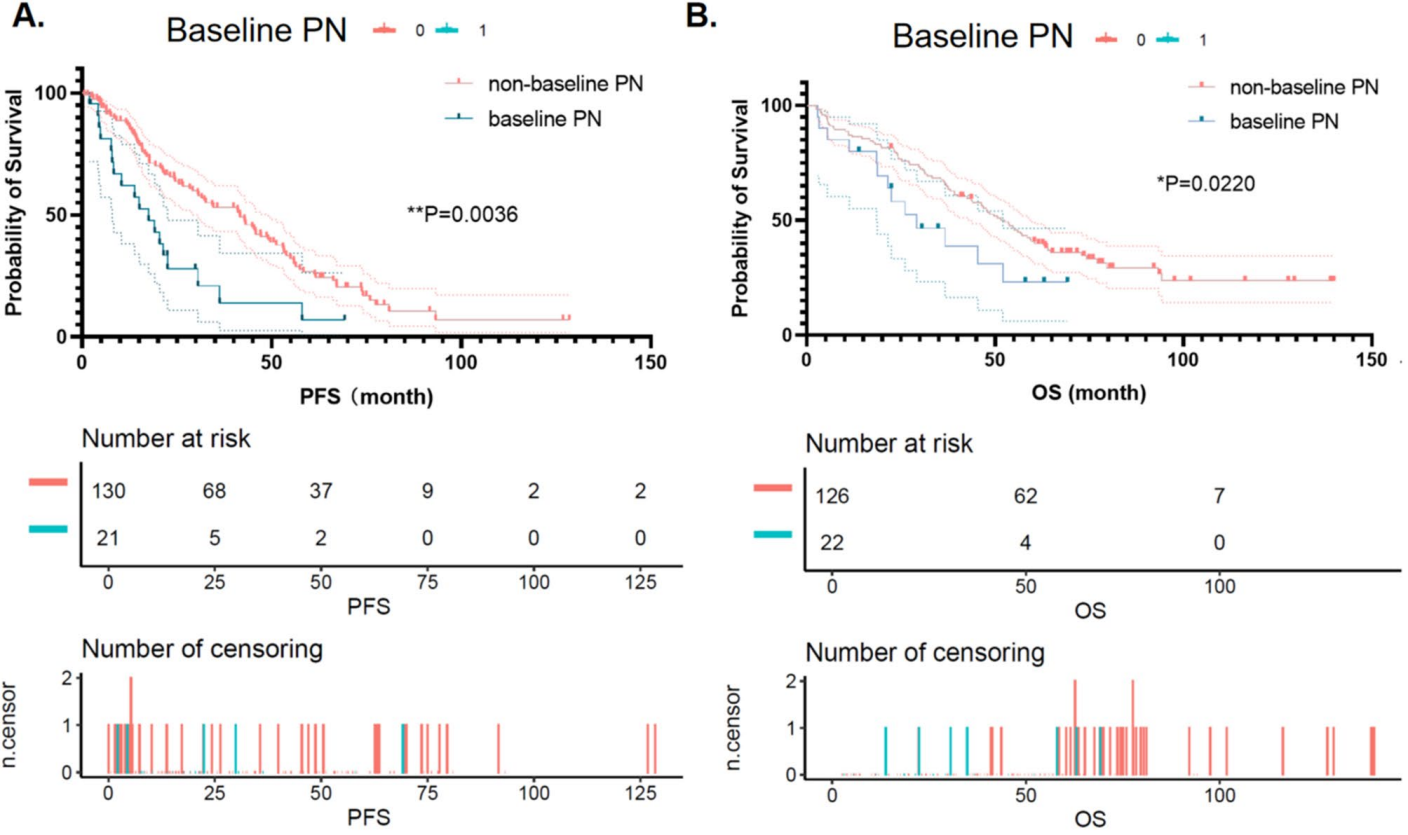
Kaplan–Meier curves (created by Empower Stats and R software, www.empowerstats.com; http://www.R-project) of progression-free survival (PFS) and overall survival (OS) according to baseline PN. (**A**) MM patients with baseline PN had poorer PFS than with non-baseline PN (*p* = 0.0036). (**B**) Compared to MM patients with baseline PN, MM patients with non-baseline PN had better OS (*p* = 0.0220). *MM* multiple myeloma, *PN* peripheral neuropathy, *PFS* progression-free survival, *OS* overall survival.

Furthermore, to observe the prognosis of patients, data of PFS and OS of MM patients until death or loss to follow-up were recorded. The K-M curve showed that patients with baseline PN had worse PFS (*p* = 0.0036, Fig. 5A) and OS (*p* = 0.0220, Fig. 5B). After adjusting age, gender, BMI, immunoglobin type, light chain type, DS stage, Creatinine, β2-MG, LDH, bortezomib dose, and administration route, taking baseline PN as the dependent variable, results of Cox multivariate regression analysis showed that patients with baseline PN had inferior PFS (HR 2.05, 95% CI (1.17, 3.59), *p* = 0.0122) and OS (HR 1.90, 95% CI (1.04, 3.47), p = 0.0381) to patients without baseline PN (Table 3).

## Discussion

Multiple myeloma is a monoclonal plasma cell malignant hematological disease. PN is an essential complication of MM, which can be caused by MM itself and treatment^16^. However, the causes of baseline PN in MM patients are not completely clear and may be related to amyloid deposition, cytokine-mediated injury mechanisms, and direct compression of radiculopathy by tumors^11,17^.

The incidence of the baseline PN in MM patients in this study was 14.8%, which was consistent with international reports of 1–2 to 20%^18^. PN is related to the presence of monoclonal antibodies, and their grade does not need to be too high to trigger PN. Therefore, well-controlled MM patients and even monoclonal gammopathy of undetermined significance (MGUS) patients may develop PN^19^. Many factors affect PN in MM patients. Studies have shown that BMI is related to PN in MM patients. Compared with non-obese patients, obese patients have a higher risk of developing BiPN^20^. Vitamin D may also play a role in the development of PN in MM patients^10^. The affecting brain-derived neurotrophic factor (BDNF) is a nerve growth factor that plays an essential role in maintaining the nervous system. Therefore, the grade of BDNF protein may be a tool for Chemical-induced peripheral neuropathy risk assessment^21^. However, when we collected patient information, we did not distinguish in detail whether the baseline PN of the patients was caused by the MM itself or by other reasons, which was a limitation of the article. Our research showed that the age of MM patients was an independent factor of baseline PN. Especially, patients over 60 years old had more severe baseline PN than those under 60 years old, and patients over 65 years old than under 65 years old. The higher the age, the greater the probability of the occurrence of baseline PN, which was consistent with the results of an observational study by Leone et al. They found that the age was associated with distal symmetrical sensory peripheral neuropathy in MM^22^.

MM is still an incurable disease. Advances in treatment strategies, such as proteasome inhibitors, autologous stem cell transplantation and immunotherapy have improved the prognosis of MM patients. However, at the same time, it has also increased the therapy-related side effects of MM patients. Bortezomib is a first-generation proteasome inhibitor. It is used in combination with other compounds and is the cornerstone of MM treatment^23^. Bortezomib preferentially targets small myelinated and unmyelinated fibers, so it usually causes hyperesthesia, neuropathic pain, and temperature sensation changes in limbs^24^. BiPN largely affects the patients’ life quality and the treatment continuity^25^. A variety of factors affect BiPN. A genome-wide association study found that four genes related to the development and signal transduction of the nervous system are related to BiPN^26^. Additionally, the dosage of bortezomib, subcutaneous or intravenous administration and other combination drugs can affect the degree of BiPN^27,28^. Both MM disease itself and therapy can cause PN; however, the relationship between the patients’ baseline PN and BiPN is unclear. Our study showed that MM patients with baseline PN have more severe BiPN and worse PN outcomes than MM patients without baseline PN. This suggested that when choosing treatment for MM patients with baseline PN, we need to pay more attention to the the patients’ PN grade, timely application of neuroprotective therapy, and adjustment of treatment plans. The diagnosis of PN in our research was mainly based on the clinical manifestations of the patients, physical examination and electromyography. Given the lack of sensitive diagnostic endpoints for chemotherapy-associated peripheral neuropathy (CIPN), recent efforts showed that corneal confocal microscopy could detect CIPN, which couldn’t be monitored by traditional neuropathy detection methods^29,30^. Further objective and sensitive diagnostic and assessment systems for PN are expected.

When treating MM patients, effectiveness and prognosis are also the concerned issues. The cytogenetic characteristics of monoclonal plasma cells, the DS or (R)ISS stage at the initial stage of diagnosis, the treatment plan, autologous stem cell transplantation and other factors affect the effectiveness and prognosis of the patient^31–33^. Our results indicated that patients with baseline PN had worse PFS and OS. Our research suggested that patients with baseline PN were often older and were treated with reduced-dose of bortezomib-based regimens. These factors can also affect the prognosis of patients. Therefore, multivariate regression and K-M curve analysis after the adjustment of potential factors were implemented, and the results showed that the baseline PN was significantly independently correlated with PFS and OS. It may be a clue implying that the basic peripheral nerve compression capacity, degree of amyloid deposition and microenvironment in MM patients are not conducive to the clinical outcomes of patients. Of course, further physiological and pathological mechanisms remain to be explored.

Our findings are based on a single-center retrospective observational study, which has certain limitations. Some patients were discarded due to incomplete data, which may have a certain selection bias; in the multivariate regression study, although we adjusted some factors that may affect the outcome, there were still some confounding factors that we have not explored. Additionally, sample size was small in this study, and a prospective study involving multiple centers with more participants are needed to further verify our findings.

## Conclusion

This study showed that the baseline PN of newly diagnosed MM patients is independently related to the age at the time of onset. Older MM patients suffered more severe baseline PN. MM patients with baseline PN had more severe BiPN and worse PN outcome. Additionally, patients with baseline PN had worse PFS/OS than patients without baseline PN.

## Supplementary Information

Supplementary Table 1.

## Data Availability

The datasets used and/or analysed during the current study available from the corresponding author on reasonable request.
