# Supplementary material for: Baseline peripheral neuropathy was associated with age and a prognostic factor in newly diagnosed multiple myeloma patients
Source: Sci Rep. 2022 Jun 16;12:10061. doi: 10.1038/s41598-022-13935-2 (PMC9203796; doi:10.1038/s41598-022-13935-2)
Supplement: Supplementary file 1 — Supplementary Table 1. [file 41598_2022_13935_MOESM1_ESM.pdf]

Nervous system disorders					
	Grade				
**Adverse Event**	**1**	**2**	**3**	**4**	**5**
Paresthesia	Mild symptoms	Moderate symptoms; limiting instrumental ADL	Severe symptoms; limiting self care ADL	-	-
Definition: A disorder characterized by functional disturbances of sensory neurons resulting in abnormal cutaneous sensations of tingling, numbness, pressure, cold, and warmth that are experienced in the absence of a stimulus.					
Peripheral motor neuropathy	Asymptomatic; clinical or diagnostic observations only; intervention not indicated	Moderate symptoms; limiting instrumental ADL	Severe symptoms; limiting self care ADL; assistive device indicated	Life-threatening consequences; urgent intervention indicated	Death
Definition: A disorder characterized by inflammation or degeneration of the peripheral motor nerves.					
Peripheral sensory neuropathy	Asymptomatic; loss of deep tendon reflexes or paresthesia	Moderate symptoms; limiting instrumental ADL	Severe symptoms; limiting self care ADL	Life-threatening consequences; urgent intervention indicated	Death
Definition: A disorder characterized by inflammation or degeneration of the peripheral sensory nerves.					
Phantom pain	Mild pain	Moderate pain; limiting instrumental ADL	Severe pain; limiting self care ADL	-	-
Definition: A disorder characterized by marked discomfort related to a limb or an organ that is removed from or is not physically part of the body.					

S-Table 1. The grade of peripheral neuropathy (PN) from CTCAE_4.03. CTCAE, common terminology criteria for adverse events. ADL, activities of daily living.
